# Using electronic patient records: defining learning outcomes for undergraduate education

**DOI:** 10.1186/s12909-019-1466-5

**Published:** 2019-01-22

**Authors:** S. K. Pontefract, K. Wilson

**Affiliations:** 1 School of Pharmacy, College of Medical and Dental Sciences, University of Birmingham, Institute of Clinical Sciences, Birmingham, B15 2TT UK; 2 University Hospitals Birmingham NHS Foundation Trust, Edgbaston, Birmingham, B15 2SP UK; 3 Manchester Medical School, Faculty of Biology, Medicine and Health, University of Manchester, Manchester, M13 9PL UK

**Keywords:** Medical education, Undergraduate, Electronic patient record, Electronic prescribing

## Abstract

**Background:**

Healthcare professionals are required to access, interpret and generate patient data in the digital environment, and use this information to deliver and optimise patient care. Healthcare students are rarely exposed to the technology, or given the opportunity to use this during their training, which can impact on the digital competence of the graduating workforce. In this study we set out to develop and define domains of competence and associated learning outcomes needed by healthcare graduates to commence working in a digital healthcare environment.

**Method:**

A National Working Group was established in the UK to integrate Electronic Patient Records (EPRs) into undergraduate education for healthcare students studying medicine, pharmacy, nursing and midwifery. The working group, comprising 12 academic institutions and representatives from NHS England, NHS Digital and EPR system providers, met to discuss and document key learning outcomes required for using EPRs in the healthcare environment. Outcomes were grouped into six key domains and refined by the group prior to external review by experts working in medical education or with EPRs.

**Results:**

Six key domains of competence and associated learning outcomes were identified and defined. External expert review provided iterative refinement and amendment. The agreed domains were: 1) Digital Health: work as a practitioner in the digital healthcare environment; 2) Accessing Data: access and interpret patient data to inform clinical decision-making; 3) Communication: communicate effectively with healthcare professionals and patients in the digital environment; 4) Generating data: generate data for and about patients within the EPR; 5) Multidisciplinary working: work with healthcare professionals with and alongside EPRs; and 6) Monitoring and audit: monitor and improve the quality and safety of healthcare.

**Conclusion:**

The six domains of competence and associated learning outcomes can be used by academics to guide the integration of EPRs into undergraduate healthcare programmes. This is key to ensuring that the future healthcare workforce can work with and alongside EPRs.

**Electronic supplementary material:**

The online version of this article (10.1186/s12909-019-1466-5) contains supplementary material, which is available to authorized users.

Stopford building.

## Introduction

The patient record is fast becoming digitised. Electronic Patient Records (EPRs) enable real-time sharing of information within and across the interface of care. Digital interventions within the EPR such as electronic prescribing (ePrescribing), or Computerised Physician Order Entry (CPOE), can reduce the risk of error [1–4]. A reduction in risk translates to improved patient safety and potential cost savings for the National Health Service (NHS), with interoperability of systems offering further reductions in expenditure [5]. Owing to the proven benefits of the technology, NHS England (NHSE) has committed to making all patient care records digital, real-time and interoperable by 2023 through the creation and integration of EPRs [6].

The effective utilisation of EPRs relies on information technology (IT) skills, user familiarity, competence, and a knowledge of data within systems to effectively inform clinical decision-making. Training is essential for the successful implementation and on-going use of the technology [7, 8]; sub-optimal use can increase the risk of clinical and procedural errors [9–11]. Lack of training, education and staff development in this area have been identified as major barriers to innovation [12]. It could be argued that such training of healthcare professionals has not kept pace with digital challenges to date [13]. NHS England is working to improve the digital competence of the workforce through the introduction of digital academies and clinical information officers [14]. This will transform care by encouraging staff to embrace new technology, however the training does not include the future workforce—healthcare students. Students are increasingly exposed to EPR systems, and so need to be given the opportunity to develop the competencies to *“access, discriminate, analyse, apply knowledge and master large flows*” of information from these [13]. Importantly, they require robust training within an environment made safe for learning. This research aimed to develop and define competencies needed by healthcare graduates to commence working in a digital healthcare environment. Learning outcomes were selected to be identified since these are used throughout undergraduate and postgraduate healthcare training in the United Kingdom (UK) [15, 16]. As such, medical educators are familiar with how the roles defined within a competency can be translated into outcomes as knowledge, attitudes and skills and used to monitor the progress of students and trainees [17, 18].

## Method

A National Working Group of academics in the UK was established to integrate EPRs into the undergraduate healthcare education they oversee. The group comprised academics working in medicine, pharmacy, nursing, midwifery and health informatics programmes across 12 different institutions along with a medical and pharmacy student. The academics were joined by representatives from NHS England, Health Education England and EPR system providers (Table 1).

**Table 1:** Demographics of working group members involved in the development of the domains of competence and learning outcomes

Profession	Specialty	Employer
Director	Analytics	EPR System supplier
Doctor	Clinical pharmacology	Academic institution
Doctor/ lecturer	Medical education and prescribing	Academic institution & General Practice
Doctor/lecturer	Clinical pharmacology	Academic institution & NHS hospital
Doctor/lecturer	Endocrinology	Academic institution & NHS hospital
Educationalist	Technology enhanced learning	Health Education England
Engineer	Research software engineering	Academic institution
Lecturer	Clinical communication	Academic institution
Lecturer	Clinical information systems	Academic institution
Lecturer	Medical education	Academic institution
Lecturer	Informatics and telematics in healthcare	Academic institution
Manager	Clinical safety	EPR System supplier
Medical student	Third year	Academic institution
Pharmacist	Digital technology	NHS England
Pharmacist	Electronic Prescribing	NHS Hospital
Pharmacist	Electronic prescribing	EPR System supplier
Pharmacist	Electronic prescribing	EPR System supplier
Pharmacist	Curriculum development	Academic institution
Pharmacist /lecturer	Prescribing	Academic institution
Pharmacist/ lecturer	Prescribing	Academic institution
Pharmacist/ lecturer	Medication safety	Academic institution
Pharmacist/ lecturer	Pharmacy practice	Academic institution
Pharmacist/lecturer	Electronic patient records and medication errors	Academic institution
Pharmacy student	Third year	Academic institution
PhD student	Electronic patient records	Academic institution

The group were asked to discuss and document competencies and associated learning outcomes that they considered to be needed by their healthcare students, as they learned with and alongside EPRs in modern healthcare settings. A competency was defined to the group as “an observable behaviour in the context of the role of the healthcare professional”. The group were asked to consider the learning outcomes needed by graduates to meet the needs of patients and other healthcare professionals in the safe and effective delivery of care [19]. The group were aware that no such learning outcomes currently existed in undergraduate healthcare programmes, although many professional bodies were beginning to include their use in professional standards (e.g. Royal Pharmaceutical Society professional standards for hospital pharmacy services include a section on ‘Digital technology and informatics to support medicines use’) [20]. Upon completion of a first draft, the learning outcomes were grouped into six overarching domains of competence by two academics (SP, KW) and presented back to the group for review and refinement. The group agreed upon the wording for each domain of competence and allocation and wording of learning outcome to create a final draft (Additional file 1). This was written up and disseminated via email to the group for any final comments for refinement prior to external review.

Experts working in medical education or as healthcare professionals working with, or researching EPRs, were invited to participate in a two-round eDelphi to independently review the domains of competence and associated learning outcomes to gain consensus. Ten experts were identified through recommendations from members of the working group. The experts were asked to review the domains and outcomes developed by the working group and to make suggestions for refinement, addition, amendment or removal through electronic return of a standardised pro-forma. Following completion of the first round, competencies and learning outcomes were amended in line with feedback and sent back to participants for further review, with any other comments and suggestions included in the final document for publication (Additional file 2).

## Results

The working group agreed upon six domains of competence and 29 learning outcomes related to the training of undergraduate healthcare students in the context of EPRs (Additional file 1). The final list was emailed to eight experts who agreed to participate in the eDelphi process (Table 2).

**Table 2:** Demographic details of the eight participants who took part in the eDelphi process

Profession	Specialty	Employer
Pharmacist	Medication safety/electronic prescribing	NHS Hospital
Pharmacist	Medication safety/electronic prescribing	NHS Hospital
Professor	Workplace learning	Academic institution
Pharmacist	Clinical education and training	NHS Hospital
Doctor / lecturer	Medical education	Academic institution
lecturer	Medication education	Academic institution
Pharmaceutical safety specialist	Human factors	Industry
Doctor/lecturer	Medication errors/Electronic prescribing	Academic institution

**Fig. 1:**
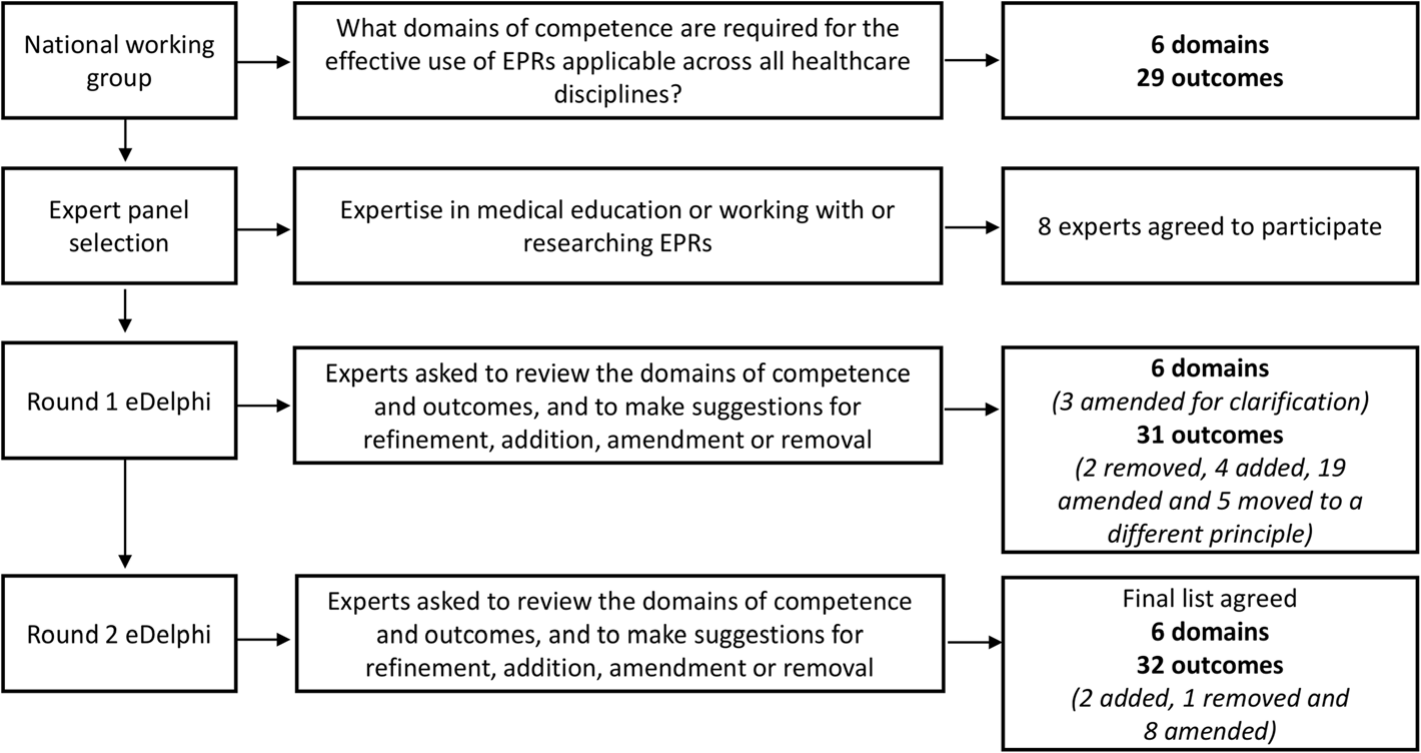
Flow chart to show the eDelphi process

In Round 1 the eDelphi process, participants made recommendations to remove two learning outcomes, add four, amend 19 and move five (See Fig. 1). Suggestions were made to remove learning outcomes that were not considered necessary, were unclear or were covered elsewhere. For example ‘*Describe the digitisation in the NHS*’ was suggested to be removed as this was not considered important for the domain of competence relating to the effective use of EPRs. Amendments were suggested to make the description of competencies and learning outcomes clearer. For example, Domain 1 ‘Digital Literacy: proficiency in the use of EPRs and adaptive to changes in this technology’, was suggested to be changed to ‘Digital Health: working as a practitioner in the digital NHS’. Digital literacy was considered to refer more to the terminology used in the context of EPRs, rather than the use of the technology. The responses from Round 1 were discussed by two academics (SP, KW), who worked to resolve any conflicting opinions and amended the domains of competence and associated learning outcomes accordingly for disseminating in Round 2.

All eight participants completed Round 2 of the process. In this round, suggestions were made to add two learning outcomes, remove one and amend 8 eight (see Fig. 1 and Additional file 3). For example, in this round one participant suggested that the reporting of adverse events should be included in ‘Monitoring and audit’ (Domain 6). Again, responses from Round 2 were discussed by two academics (SP, KW). Following two rounds of review (Fig. 1), the six domains of competence and 32 learning outcomes were agreed (see Table 3). The domains agreed were: 1) Digital health; 2) Accessing data; 3) Communication; 4) Generating data; 5) Multidisciplinary working; and 6) Monitoring and audit.

**Table 3:** Domains of competence and associated learning outcomes for undergraduate healthcare students

Domain of competence	Learning outcome	
1. Digital Health*Work as a practitioner in the digital healthcare environment.*	1.1	Outline the risks and benefits of digitisation of patient records for patients and their carers and healthcare staff.
	1.2	Describe EPR technology in different sectors of care*.
	1.3	Explain potential limitations of EPR systems and how these may impact on care.
	1.4	Explain how EPRs can facilitate workflow and the prioritisation and coordination of care within the multi-disciplinary team.
	1.5	Explain the importance of information governance and data protection in the context of EPRs†.
	1.6	Outline own responsibilities in responding to clinical decision support software*.
	1.7	Maintain accountability for your own actions in the digital environment.
2. Accessing Data:*Access and interpret patient data to inform clinical decision-making.*	2.1	Access electronic data within a healthcare setting and at the interface of care.
	2.2	Plan and review clinical care and make decisions with reference to electronic data accessed within the EPR.
	2.3	Assess accuracy of data and identify gaps to determine completeness of documentation.
	2.4	Demonstrate respect of patient consent, privacy and confidentiality when accessing data.
	2.5	Demonstrate awareness of professional responsibilities with respect to protecting appropriate access to data.
3. Communication: *Communicate effectively with healthcare professionals and patients in the digital environment.*	3.1	Apply appropriate digital terminology when documenting within the EPR.
	3.2	Document information relating to the management of patients.
	3.3	Document information for patients and their carers relating to their management.
	3.4	Communicate effectively with other healthcare professionals in the electronic environment.
	3.5	Communicate requests for tests and investigations with or to the appropriate recipient.
	3.6	Communicate with the appropriate person(s) when care needs escalating.
	3.7	Communicate effectively at the interface of care.
	3.8	Maintain patient engagement when using the EPR system.
4. Generating data:*Generate data for and about patients within the EPR.*	4.1	Account for the necessity of the data you generate.
	4.2	Demonstrate respect of patient consent, privacy and confidentiality when generating data.
	4.3	Generate data that is necessary and complete.
	4.4	Review, manage and document treatment plans.
	4.5	Document the prescribing, dispensing or administration of medicines for patients within the duties of your profession, according to legal and good practice requirements†.
5. Multidisciplinary working:*Work with healthcare professionals with and alongside EPRs.*	5.1	Demonstrate respect for professional identity, roles and requirements from the system when working with other healthcare professionals.
	5.2	Demonstrate effective coordination of care within and across healthcare teams.
	5.3	Demonstrate shared decision-making with other healthcare professionals in the context of the EPR.
6. Monitoring and audit: *Monitor and improve the quality and safety of healthcare.*	6.1	Use patient and prescription data to support monitoring and audit for quality improvement.
	6.2	Escalate and report concerns about the function or capability of the EPR system identified through monitoring.
	6.3	Document adverse drug reactions and report these using the EPR as necessary.
	6.4	Respect research ethics in the use of data captured from the EPR.

## Discussion

Training is a key for the successful implementation of technology in the healthcare environment [21], and insufficient training can lead to sub-optimal use. In this study we set out to develop and define competencies and associated learning outcomes needed by healthcare graduates to commence working in a digital healthcare environment. A number of professionals were involved in the iterative development of six domains of competence and 32 learning outcomes, which have been identified as integral to the training of undergraduate healthcare students to work with and alongside EPRs. These provide the baseline knowledge for the use of EPR technology in healthcare, as well as the essential skills and professional attitude to work alongside technology to provide patient care.

The first domain of competence, ‘Digital health’, ensures that students have an understanding of the technologies available, the impact they can have on clinical care, and how to work safely with and alongside the systems. Healthcare students may be aware, for example, that EPRs such as CPOE can reduce the risk of medication errors [22], but may be less cognisant of how to respond to clinical decision support or the complex functionality of systems to optimise workflow and coordinate care. Learning outcomes relating to ‘Accessing data’ ensure the healthcare student can demonstrate effective access and interpretation of patient data to inform clinical decision-making. Information held within EPRs can be fragmented when compared to paper-based records; this fragmentation may have an impact on clinical reasoning [23]. It is important that healthcare staff can access relevant data in order to build a patient story and make clinical decisions about patient care [24].

It is important that staff consider the integrity of data in systems. Incorrect information within systems, through wrong data entry or miscommunication, can lead to medical error [25]. Healthcare students need the awareness, skills and experience to deal with issues novel to the electronic environment when generating data. Although technology such as CPOE can reduce the risk of certain error types and cost-savings, research has also shown that digitising the patient record can change communication, coordination of work and workflow patterns [26–31]. Medication errors caused by staff interacting and generating data within electronic prescribing systems can introduce new risks to patient safety [9, 31, 33]. For example, new error types such as those created from selecting the wrong patient or wrong drug [32, 34].

The delivery of patient care is dependent on effective communication between healthcare staff and between staff and patients. Failures in the process of communication are one of the leading causes of adverse events in healthcare [7, 35]. Where communication has traditionally been undertaken through verbal and written forms, the digital environment now offers new and exciting ways to augment these processes. It is important that students know how to communicate effectively within the EPR and know when to adjust their modality of communication according to the situation [36]. Training related to EPR communication has also been shown to improve history taking skills and empathetic engagement in patient care [37].

Within modern healthcare, multidisciplinary teamwork is the norm. This means that access and contribution to the EPR is relevant to all. It is important that different professions across the healthcare team understand each other’s requirements from the system. This collaborative approach needs to extend beyond respect and communication. Users of the EPR across professions must contribute to the generation and review of data, and embrace the coordination of care and sharing of decisions.

Finally, the increasing use of digital records means large volumes of digital data are being generated [38], which can be used to drive quality improvement in healthcare [39]. The learning outcomes outlined for ‘Monitoring and audit’ ensure that students can effectively capture and interpret data, and can demonstrate respect for the ethical considerations in relation to this type of research.

The domains of competence and associated learning outcomes developed provide an overview of the knowledge, skills, and attitudes needed by healthcare graduates to commence working in a digital healthcare environment. In the same way that healthcare is dynamic and non-linear, many of these learning outcomes will relate to each other, and may not be measurable in isolation. For example, under Communication, ‘Document information relating to the management of patients’ overlaps with learning outcomes in Domain 4 for ‘Generating data’. Similarly, under Communication, ‘Communicate effectively with other healthcare professionals in the electronic environment’ overlaps with ‘Demonstrate effective coordination of care within and across healthcare teams’.

The six domains of competence and associated learning outcomes outlined from this study are designed for use by academics to guide the integration of EPRs into undergraduate healthcare programmes. It has been argued that changes to healthcare curricular (particularly in medical education) can follows *“fads”* and that changes need to be appropriately evidenced for inclusion since many are already working at capacity [19]. The evidence presented here clearly shows that education with and alongside EPRs is fundamental to the future practice of healthcare professionals and the safe and effective delivery of care in the twenty-first Century. However, the integration of the technology into teaching can be gradual process. Kushniruk et al. (2009) describe two approaches to this: the first is “loose coupling”, where the EPR is demonstrated to students and assignments involve the EPR outside of the classroom, and the second is “tight coupling” where the EPR is fully integrated into teaching, assignments and assessment [40]. The “continuum” as it is described demonstrates how integration of the EPR into education can be varied and on-going process of development and refinement. Importantly, the learning outcomes defined can be used to guide the development of educational initiatives along this continuum.

The implementation of the learning outcomes into curricular requires the technological resource to facilitate delivery—that is the EPR technology for educators to implement for students to interact with. This is likely to be a barrier for many academic institutions. The researchers have been working with an EPR system provider to create a University simulation EPR [41] so that the education may be delivered through didactic, simulated, experiential and reflective pedagogy with and alongside the EPR.

### Strengths and limitations

A number of experts were involved with the development of the domains of competence and associated learning outcomes, who worked in a range of settings including medical education, informatics or healthcare, or a combination of these roles. In addition, two students were present to ensure all the various stakeholders were represented. This was important to ensure that the learning outcomes developed were relevant and could be successfully implemented into undergraduate curricula. The participants for the eDelphi were purposively selected based on their experience working in medical education and/or medication safety/electronic patient records. Although this had the potential to introduce bias and affect the quality of data generated, the researchers (SP and KW) compiled all the feedback and the final version of the domains of competence and learning outcomes were shared with the working group for comment.

Since the subject area was new, with no existing curricula to review, the researchers selected a methodology that would ensure iterative development and refinement of the learning outcomes, to ensure that the final list of would meet the needs of healthcare and meet the requirements of medical educators. However, all participants from both the working group and the eDelphi were UK-based, and therefore the results may not be entirely transferable to other countries.

Finally, as for any outcome-based educational approach, the competencies defined here will require regular review to ensure relevance to practice.

## Conclusions

The move to digitise patient records introduces new challenges for healthcare professionals and healthcare students who interact with patients. They also introduce new challenges and opportunities for the academics that provide healthcare teaching. Domains of competence and associated learning outcomes have been identified to guide the teaching of students to work with and alongside EPRs. These are important for ensuring that healthcare students gain structured experience to promote the safe, effective and optimal use of EPRs from an undergraduate level.

## Additional files

Additional file 1: Domains of competence and associated outcomes for undergraduate healthcare students developed during the working group prior to external review. (DOCX 18 kb)

Additional file 2: Feedback for amendment and refinement from Round 1 of the eDelphi process. (DOCX 23 kb)

Additional file 3: Feedback for amendment and refinement from Round 2 of the eDelphi process. (DOCX 22 kb)
