# Supplementary material for: Using electronic patient records: defining learning outcomes for undergraduate education
Source: BMC Med Educ. 2019 Jan 22;19:30. doi: 10.1186/s12909-019-1466-5 (PMC6341543; doi:10.1186/s12909-019-1466-5)
Supplement: Supplementary file 1 — Domains of competence and associated outcomes for undergraduate healthcare students developed during the working group prior to external review. (DOCX 18 kb) [file 12909_2019_1466_MOESM1_ESM.docx]

**Additional file 1**

Domains of competence and associated outcomes for undergraduate healthcare students developed during the working group prior to external review.

| **Domain of competence** | **Learning outcome** | |
| --- | --- | --- |
| **Digital Literacy**  *Proficiency in the use of EPRs and adaptive to changes in this technology.* | 1.1 | Describe the process of digitisation in the NHS |
|  | 1.2 | Explain why digitisation of healthcare is crucial for modern clinical care |
|  | 1.3 | Apply appropriate digital terminology in communication and explanation |
|  | 1.4 | Explain the safety standards that are applied in the design of UK EPRs |
|  | 1.5 | Explain how EPR technology can support clinical decision-making |
|  | 1.6 | Explain potential limitations of EPR systems |
| **Accessing data**  *Access and interpret patient data to inform clinical decision making.* | 2.1 | Plan and review clinical care with reference to electronic data contained within an EPR |
|  | 2.2 | Share data across healthcare settings, professions and where appropriate, with patients or their carers |
|  | 2.3 | Demonstrate respect of patient consent, privacy and confidentiality when accessing and sharing data |
|  | 2.4 | Demonstrate knowledge and understanding of information governance and the Data Protection Act |
| **Communication**  *Communicate effectively with healthcare professionals and patients in the digital environment.* | 3.1 | Effectively summarise and document information relating to the management of patients and with patients |
|  | 3.2 | Demonstrate effective electronic communication with other healthcare professionals |
|  | 3.3 | Communicate requests for tests and investigations |
|  | 3.4 | Communicate when care needs escalating |
|  | 3.5 | Communicate at the interface of care |
|  | 3.6 | Demonstrate a professional demeanour and maintain patient engagement when using EPR systems |
| **Generating data**  *Generate data for and about patients within the EPR.* | 4.1 | Assess accuracy and completeness of data. |
|  | 4.2 | Generate prescriptions for patients |
|  | 4.3 | Review and manage treatment |
|  | 4.4 | Maintain accountability and ongoing responsibility |
|  | 4.5 | Account for the necessity of the data you generate |
|  | 4.6 | Demonstrate respect of patient consent, privacy and confidentiality |
| **Multi-disciplinary working**  *Work with other healthcare professionals interacting via with EPRs.* | 5.1 | Demonstrate respect for professional identity, roles and requirements from the system |
|  | 5.2 | Demonstrate team work |
|  | 5.3 | Undertake shared decision-making |
|  | 5.4 | Explain how the EPR can facilitate workflow, and the prioritisation and coordination of care. |
| **Audit and monitoring**  *Monitor and improve the quality and safety of healthcare.* | 6.1 | Use patient and prescription level data to support monitoring and audit |
|  | 6.2 | Escalate concerns identified through the monitoring of EPR safety |
|  | 6.3 | Respect research ethics in the meaningful use of data |
