# Supplementary material for: Using electronic patient records: defining learning outcomes for undergraduate education
Source: BMC Med Educ. 2019 Jan 22;19:30. doi: 10.1186/s12909-019-1466-5 (PMC6341543; doi:10.1186/s12909-019-1466-5)
Supplement: Supplementary file 2 — Feedback for amendment and refinement from Round 1 of the eDelphi process. (DOCX 23 kb) [file 12909_2019_1466_MOESM2_ESM.docx]

**Additional file 2**

Feedback for amendment and refinement from Round 1 of the eDelphi process

| **Domain of competence** | **Learning outcome** | | **Suggestion for refinement/amendment** |
| --- | --- | --- | --- |
| **Digital Literacy**  Proficiency in the use of EPRs and adaptive to changes in this technology. | The domains of competence and learning outcomes are not considered related. Recommended to amend to Digital Health, and “*Working as a practitioner in the digital NHS*” | | |
|  | **1.1** | Describe the process digitisation in the NHS | Suggest removing as does not fit with the description of the domain. |
|  | **1.2** | Explain why digitisation of healthcare is crucial for modern clinical care | Suggest amending to: “Outline the benefits of digitisation for patients and their carers, and healthcare staff”. |
|  | **1.3** | Apply appropriate digital terminology in communication and explanation | Suggest moving to the ‘Communication’ domain and amend to “Apply appropriate digital terminology when documenting within the EPR”. |
|  | **1.4** | Explain the safety standards that are applied in the design of UK EPRs | Suggest removing as does not fit with the description of the domain. |
|  | **1.5** | Explain how EPR technology is a tool to facilitate clinical decision-making | Suggest amending to “Describe EPR technology in different sectors of care”. |
|  | **1.6** | Explain potential limitations of EPR systems | Suggest amending and adding at the end”…and how these may impact on care”. |
|  | Consider new learning outcome “Outline own responsibilities in responding to clinical decision support software”. | | |
|  | | | |
| **Accessing Data:**  Access and interpret patient data to inform clinical decision making | **2.1** | Plan and review clinical care with reference to electronic data contained within an EPR | Suggest amending to “Plan and review clinical care, and make decisions with reference to electronic data contained within an EPR”. |
|  | **2.2** | Share data across healthcare settings, professions and where appropriate, with patients or their carers | Suggest amending to “share” to “Access data within a healthcare setting and at the interface of care”. |
|  | **2.3** | Demonstrate respect of patient consent, privacy and confidentiality when accessing and sharing data | Suggest amending to just “when accessing data” (i.e. remove the sharing of data). |
|  | **2.4** | Demonstrate knowledge and understanding of information governance and the Data Protection Act |  |
|  | Consider new learning outcome “Demonstrate awareness of your professional responsibility to protect appropriate access to data” | | |
|  | | | |
| **Communication**  *Communicate effectively with healthcare professionals and patients in the digital environment.* | **3.1** | Effectively summarise and document information relating to the management of patients and with patients | Suggest amending to “Document information relating to the management of patients” and “Document information for patients and their carers relating to their management”. |
|  | **3.2** | Demonstrate effective electronic communication with other healthcare professionals | Suggest amending to “Communicate effectively with other healthcare professionals in the electronic environment”.  [i.e. use of “with” to ensure exchange of information]. |
|  | **3.3** | Communicate requests for tests and investigations | Suggest adding “…to the appropriate recipient” at the end. |
|  | **3.4** | Communicate when care needs escalating | Suggest amending to “Communicate with the appropriate person(s) when care needs escalating”. |
|  | **3.5** | Communicate at the interface of care |  |
|  | **3.6** | Demonstrate a professional demeanour and maintain patient engagement when using EPR systems | Suggest amending to “Maintain patient engagement when using the EPR system”. |
|  | | | |
| **Generating data**  *Generate data for and about patients within the EPR* | **4.1** | Assess accuracy and completeness of data. | Suggest moving to “Accessing data” domain, and amend to “Assess accuracy of data and identify gaps to determine completeness of the documentation”. |
|  | **4.2** | Generate prescriptions for patients | Suggest amending to “Prescribe, dispense or administer medicines for patients…” and add “…according to legal and good practice requirements”. |
|  | **4.3** | Review and manage treatment | Suggest amending to “Review, manage and document treatment plans”. |
|  | **4.4** | Maintain accountability and ongoing responsibility | Suggest moving to Domain 1 and add at the end “…in the digital environment”. |
|  | **4.5** | Account for the necessity of the data you generate | Suggest moving to Domain 1 and amend to “Generate data that is necessary and complete”. |
|  | **4.6** | Demonstrate respect of patient consent, privacy and confidentiality | Suggest add at the end “…when generating data”. |
|  | | | |
| **Multi-disciplinary working**  *Work with other healthcare professionals interacting via with EPRs* | Delete ‘other’ in the domains. | | |
|  | **5.1** | Demonstrate respect for professional identity, roles and requirements from the system | Suggest amending to “…when working with different healthcare professionals”. |
|  | **5.2** | Demonstrate team work | Suggest amending to “Demonstrate effective coordination of care within and across healthcare teams”. |
|  | **5.3** | Undertake shared decision-making | Suggest amending to “Demonstrate shared decision-making with other healthcare professionals in the context of the EPR”. |
|  | **5.4** | Explain how the EPR can facilitate workflow, and the prioritisation and coordination of care. | Suggest moving to Domain 1 and add “…within the multi-disciplinary team”. |
|  | | | |
| **Audit and monitoring**  *Monitor and improve the quality and safety of healthcare.* | Suggest amending domain to “Monitoring and Audit”. | | |
|  | **6.1** | Use patient and prescription level data to support monitoring and audit |  |
|  | **6.2** | Escalate concerns identified through the monitoring of EPR safety | Suggest amending to “Escalate and report concerns about the function or capability of the EPR system identified through monitoring”. |
|  | **6.3** | Respect research ethics in the meaningful use of data | Suggesting amending to “… captured from EPRs”. |
