# Supplementary material for: Using electronic patient records: defining learning outcomes for undergraduate education
Source: BMC Med Educ. 2019 Jan 22;19:30. doi: 10.1186/s12909-019-1466-5 (PMC6341543; doi:10.1186/s12909-019-1466-5)
Supplement: Supplementary file 3 — Feedback for amendment and refinement from Round 2 of the eDelphi process. (DOCX 22 kb) [file 12909_2019_1466_MOESM3_ESM.docx]

**Additional file 3**

Feedback for amendment and refinement from Round 2 of the eDelphi process

| **Domain of competence** | **Learning outcome** | | **Suggestion for refinement/amendment** |
| --- | --- | --- | --- |
| **Digital Health**  *Working as a practitioner in the digital NHS* | **1.1** | Outline the benefits of digitisation for patients and their carers, and healthcare staff. | Suggest amending to “Outline the risks and benefits of the digitisation of patient records...” |
|  | **1.2** | Describe EPR technology in different sectors of care. |  |
|  | **1.3** | Explain potential limitations of EPR systems and how these may impact on care. |  |
|  | **1.4** | Explain how EPRs can facilitate workflow and the prioritisation and coordination of care within the multi-disciplinary team. |  |
|  | **1.5** | Explain the importance of information governance and data protection. | Suggest adding “…in the content of EPRs”. |
|  | **1.6** | Outline own responsibilities in responding to clinical decision support software. |  |
|  | **1.7** | Maintain accountability and ongoing responsibility in the digital environment. | Suggest amending to “Maintain accountability for you own actions in the digital environment”. |
|  | Suggesting adding to Domain 1 “Explain the importance of information governance and data protection in the context of EPRs”. | | |
|  | | | |
| **Accessing Data**  *Access and interpret patient data to inform clinical decision-making* | **2.1** | Access data within a healthcare setting and at the interface of care. | Suggest amending to “Access electronic data within…” |
|  | **2.2** | Plan and review clinical care and make decisions with reference to electronic data accessed within the EPR. |  |
|  | **2.3** | Assess accuracy of data and identify gaps to determine completeness of documentation. |  |
|  | **2.4** | Demonstrate respect of patient consent, privacy and confidentiality when accessing data. |  |
|  | **2.5** | Demonstrate knowledge and understanding of information governance and data protection. | Suggest removing as overlaps between 2.4, 2.5 & 2.6. |
|  | **2.6** | Demonstrate awareness of professional responsibilities with respect to protecting appropriate access to data. |  |
|  |  |  |  |
| **Communication**  *Communicate effectively with healthcare professionals and patients in the digital environment* | **3.1** | Apply appropriate digital terminology when documenting within the EPR. |  |
|  | **3.2** | Document information relating to the management of patients. |  |
|  | **3.3** | Document information for patients and their carers relating to their management. |  |
|  | **3.4** | Communicate effectively with other healthcare professionals in the electronic environment. |  |
|  | **3.5** | Communicate requests for tests and investigations with or to the appropriate recipient. |  |
|  | **3.6** | Communicate with the appropriate person(s) when care needs escalating. |  |
|  | **3.7** | Communicate at the interface of care. | Suggest amending to “Communicate effectively at the interface of care” |
|  | **3.8** | Maintain patient engagement when using the EPR system. |  |
|  | | | |
| **Generating data**  *Generate data for and about patients within the EPR* | **4.1** | Generate data that is necessary and complete. |  |
|  | **4.2** | Prescribe, dispense or administer medicines for patients within the duties of your profession, according to legal and good practice requirements. | Suggest amending to “Document the prescribing, dispensing or administration of medicines for patients within the duties of your profession, according to legal and good practice requirements”. |
|  | **4.3** | Review, manage and document treatment plans. |  |
|  | **4.4** | Account for the necessity of the data you generate. | Suggest reorder learning outcomes and move to 4.1 so flows in order of likely activity. |
|  | **4.5** | Demonstrate respect of patient consent, privacy and confidentiality when generating data. |  |
|  | | | |
| **Multidisciplinary working**  *Work with healthcare professionals interacting via with EPRs* | **5.1** | Demonstrate respect for professional identity, roles and requirements from the system when working with different healthcare professionals. | Suggest amending to:  “Demonstrate respect for professional identity, roles and requirements from the system when working with other healthcare professionals”. |
|  | **5.2** | Demonstrate effective coordination of care within and across healthcare teams. |  |
|  | **5.3** | Demonstrate shared decision-making with other healthcare professionals in the context of the EPR. |  |
|  | | | |
| **Monitoring and audit**  *Monitor and improve the quality and safety of healthcare.* | **6.1** | Use patient and prescription data to support monitoring and audit for quality improvement. |  |
|  | **6.2** | Escalate and report concerns about the function or capability of the EPR system identified through monitoring. |  |
|  | **6.3** | Respect research ethics in the meaningful use of data captured from the EPR. | Define “meaningful use” or amend to avoid use of term. |
|  | Consider adding reporting of adverse events in Domain 6. | | |
